# Impact of polygenic risk score (PRS) for coronary artery disease on physician decision-making and patient care

**DOI:** 10.3389/fgene.2025.1625822

**Published:** 2025-07-25

**Authors:** Georgios Ntritsos, Erez Ornan, Nir Gamliel, Gil Chernin, Arnold Pallay, Michael Chen, Efi Kessous, Eran Feldhay

**Affiliations:** ^1^Department of Economics, School of Economics and Management Sciences, University of Ioannina, Ioannina, Greece; ^2^ OpenDNA, Haifa, Israel; ^3^Faculty of Medicine, Hebrew University of Jerusalem, Rehovot, Israel; ^4^ Consensus Medical Group/Atlantic Health System, Montville, NJ, United States

**Keywords:** polygenic risk score (PRS), coronary artery disease (CAD), cardiovascular risk assessment, personalized medicine, genetic risk stratification, clinical decision-making, patient awareness, statin therapy

## Abstract

**Introduction:**

Polygenic risk scores (PRS) have emerged as a promising tool for refining cardiovascular risk prediction, yet their real-world impact on physician decision-making remains unclear. This study aimed to evaluate how the integration of a coronary artery disease (CAD) PRS with traditional clinical risk factors influences physician management strategies.

**Methods:**

We conducted a multicenter, prospective, open-label pilot study across three clinical sites. A total of 150 patients (aged 18–55 years, LDL-C ≤130 mg/dL, no history of diabetes or coronary artery disease) were recruited. Buccal swabs were collected for PRS analysis, and results were integrated with clinical data to generate personalized risk profiles. Physicians utilized these profiles during consultations and completed structured feedback surveys assessing PRS influence on their clinical decisions.

**Results:**

PRS findings impacted clinical decision-making in 67% of cases (100 participants). The most frequent physician response was raising patient awareness and offering patient education (73 cases), while emphasizing PRS as a tool for risk communication. In 4 specific cases, PRS findings led to new statin recommendations, while 23 cases resulted in other management modifications, including lifestyle adjustments and increased risk monitoring.

**Discussion:**

These findings highlight the potential of PRS in enhancing risk communication and clinical decision-making, primarily by reinforcing patient awareness rather than directly by altering pharmacologic management. Further research is needed to optimize PRS implementation and assess its long-term clinical impact.

## 1 Introduction

Atherosclerotic cardiovascular disease (ASCVD) is a leading cause of morbidity and mortality, affecting approximately 4.6 million individuals in the United States (Arnett et al., 2019). Coronary artery disease (CAD), as the most common form of ASCVD, is characterized by the accumulation of atherosclerotic plaque in the coronary arteries, leading to myocardial infarction, angina, and other complications (Chan and Ramji, 2022).

While the exact etiology remains uncertain, several modifiable and non-modifiable risk factors have been identified, including high cholesterol, hypertension, smoking, insulin resistance, diabetes, obesity, sedentary lifestyle, age, and dietary habits. Although genetic predisposition and family history cannot be altered, a better understanding of and patient awareness of these risk factors can facilitate the adoption of preventive measures and promote healthier lifestyle choices (Said et al., 2019).

To better quantify risk, the American College of Cardiology introduced the Pooled Cohort Equations risk score, a widely used tool that estimates the 10-year probability of major cardiovascular events, including myocardial infarction and stroke (Goff et al., 2013). This tool incorporates variables such as age, sex, race, cholesterol levels, blood pressure, medication use, diabetes status, and smoking history. More recently, in 2023, the American Heart Association introduced the PREVENT risk tool, which incorporates additional clinical parameters, including body mass index (BMI), hemoglobin A1c, albuminuria, and estimated glomerular filtration rate (eGFR), offering a more comprehensive approach to cardiovascular risk assessment (Khan et al., 2023). However, while both models offer valuable insights, they do not account for genetic predisposition, a factor that may offer additional insights into coronary artery disease susceptibility and also refine individualized risk assessment (Hadley et al., 2021).

Polygenic risk scoring (PRS) is an innovative approach to estimating an individual’s genetic predisposition to traits or diseases based on genome-wide association study (GWAS) data (Choi et al., 2020; Lambert et al., 2019; Torkamani et al., 2018). By analyzing a patient’s genotype, PRS provides a probabilistic estimate of CAD risk, complementing conventional utilized clinical risk factors. The integration of PRS into coronary risk assessment frameworks has the potential to improve risk stratification and support personalized preventive strategies for physicians and their patients.

To address the need for a more comprehensive risk assessment framework, we developed an artificial intelligence (AI)-driven platform that integrates PRS with conventional CAD risk factors. Using machine learning techniques, we incorporated genetic, clinical, and lifestyle data to develop predictive models aimed at guiding treatment decisions, enhancing patient education, in order to and optimize coronary risk management. The software employs widely recognized algorithms, including PLINK (Purcell et al., 2007; Chang et al., 2015) and PRS-CS (Ge et al., 2019), to compute polygenic scores while accounting for linkage disequilibrium, allowing for a more comprehensive evaluation of genetic risk factors in complex diseases.

This study aims to evaluate whether an AI-generated CAD risk profile that integrates PRS with traditional coronary risk factors can realistically influence physician decision-making regarding both statin therapy prescription and dosage adjustments, while also enhancing patient education on coronary artery disease prevention.

## 2 Methods

### 2.1 Study design

A multicenter, prospective, open-label pilot study was designed to assess the impact of integrating a PRS for CAD with traditional clinical risk factors on physician decision-making regarding statin therapy and patient education.

The study was conducted across three clinical sites: Cardiac Associates (Rockville, MD), Changebridge Medical Associates, a member of Consensus Health (Montville, NJ), and Montgomery Medical Clinic (Gaithersburg, MD). Participating physicians were practicing clinicians affiliated with these sites, including two Primary Care physicians and one Cardiologist. Physicians had unrestricted access to PRS-based risk profiles in real-time, following an open-label approach without blinding or randomization.

### 2.2 Study population

Participants were recruited from the three clinical sites by treating physicians based on predefined inclusion and exclusion criteria.

Inclusion criteria required participants to be adults aged 18–55 years with LDL-cholesterol (LDL-C) levels ≤130 mg/dL and a 10-year CAD risk score of ≤20%, classifying them as low to intermediate risk. Eligible participants were also required to have no prior history of diabetes mellitus or CAD. Additionally, all participants had to provide written informed consent prior to enrollment.

Exclusion criteria included pregnancy and inability to provide informed consent. Participants with a documented history of CAD—defined as prior myocardial infarction, coronary artery revascularization (either coronary artery bypass grafting or percutaneous coronary intervention), or a confirmed CAD diagnosis based on ICD-10 codes (I20.X, I21.X, I22.X, I23.X, I24.1, I25.2, I25.8, I25.9, I25.1X)—were excluded. Additional exclusion criteria included a history of stroke, transient ischemic attack, or peripheral arterial disease, as well as chronic kidney failure or diagnosed types I or II diabetes mellitus.

Physicians screened patients for eligibility using electronic health records (EHRs) and in-person assessments. Eligible participants received a detailed explanation of the study, had the opportunity to ask questions, and provided written informed consent before enrollment.

### 2.3 Polygenic risk score calculation

The PRS for CAD was derived from a large-scale multiethnic GWAS meta-analysis of approximately 9.4 million genetic variants, including 68K CAD cases and 117K controls (Nikpay et al., 2015). It was computed as a weighted sum of risk alleles at single-nucleotide polymorphisms (SNPs), with weights assigned based on the effect size estimates (beta coefficients) reported in this GWAS.

PRS calculations were performed using established computational tools, including PLINK and PRS-CS, which account for linkage disequilibrium and optimize polygenic risk estimation. To improve risk assessment, PRS values were integrated with conventional CAD risk factors, including cholesterol levels, blood pressure values, smoking status, medication usage, and reported diabetes status, in order to generate a comprehensive cardiovascular risk profile.

### 2.4 Intervention

Participants provided a buccal swab sample, which was processed at Gene by Gene, a laboratory accredited under the Clinical Laboratory Improvement Amendments (CLIA) and the College of American Pathologists (CAP), located in Houston, Texas. Genotyping was conducted using a high-density SNP array, and the resulting genetic data was securely transmitted to OpenDNA, where PRS for CAD was calculated.

**FIGURE 1:**
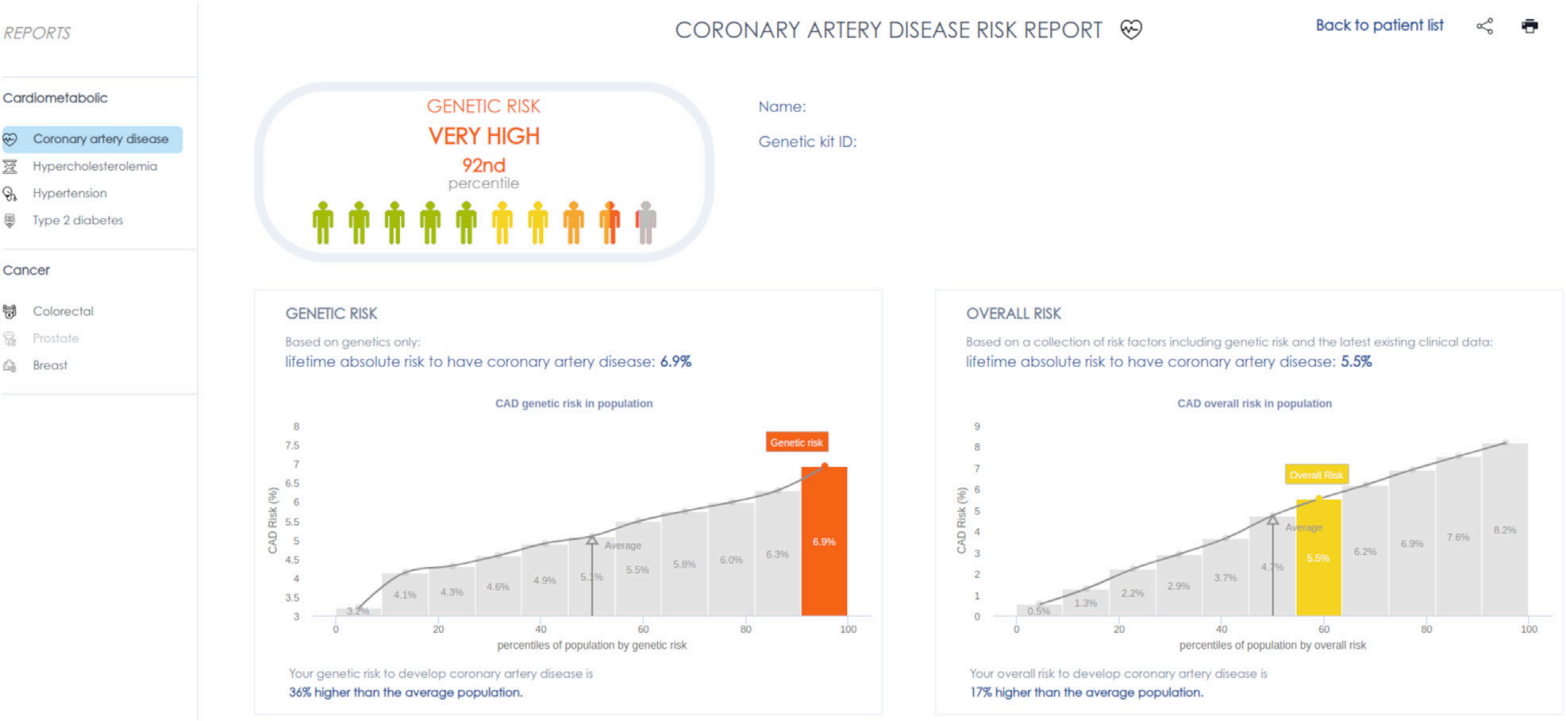
Screenshot of the clinician-facing dashboard displaying polygenic and integrated cardiovascular risk estimates.

The OpenDNA AI-driven platform automatically integrated PRS results with clinical parameters and displayed the data on an interactive dashboard, allowing physicians to access risk profiles via a secure, password-protected portal. The PRS was presented as a visual risk score on a standardized scale, embedded alongside conventional cardiovascular risk factors (e.g., LDL-C, blood pressure, smoking status), to facilitate intuitive interpretation. This integration aimed to improve usability and enhance physician engagement with genetic information in daily practice.A representative screenshot of the dashboard is provided in Figure 1. The interface presents two key risk estimates: (1) the genetic-only lifetime risk, derived exclusively from the PRS, and (2) the integrated absolute lifetime risk, which incorporates both genetic and the most recent clinical data. Both scores are displayed using a color-coded gauge that situates an individual’s risk relative to population norms. This dual presentation is intended to enhance clarity and promote informed discussion between physician and patient.

After reviewing the PRS-enhanced risk profile, physicians documented their experience through a structured feedback survey, which assessed the ease of use and the clinical relevance of PRS integration in patient care.

### 2.5 Outcome measures and data collection

The primary outcomes of this study included physician-reported impact of PRS on clinical decision-making, particularly its influence on the initiation, discontinuation, or dosage adjustment of statin therapy. Additionally, changes in physician-reported confidence in CAD risk stratification and patient education were assessed.

The secondary outcomes focused on the feasibility of PRS implementation, as well as physician perceptions regarding its usefulness in clinical workflows. Another key secondary outcome was the feasibility of integrating PRS-based risk scores into routine clinical workflows.

Data collection consisted of two primary components. The first component involved generating patient risk profiles using PRS data and integrating them with electronic health record (EHR)-derived clinical information. These reports were securely stored on OpenDNA’s platform and were accessible only to study-authorized physicians.

The second component involved physician feedback surveys, which captured insights regarding the usability and impact of PRS-based risk profiles in clinical decision-making. These surveys were completed electronically and stored securely in a restricted-access database for subsequent analysis. Physicians were asked to specify whether and how the PRS influenced their clinical decision-making, including statin prescription, lifestyle recommendations, and monitoring plans. Their responses were systematically categorized for analysis.

To ensure patient confidentiality and data security, no personal identifiers were transmitted to OpenDNA at any stage of the study. Instead, barcoded labels were used to de-identify samples and link genetic and clinical data. This method ensured that patient identities remained protected, in full compliance with HIPAA regulations and ethical guidelines.

### 2.6 Statistical analysis

Descriptive statistics were used to summarize participant demographics and physician-reported outcomes. Continuous variables were reported as means and standard deviations, while categorical variables were presented as frequencies and proportions.

Given the exploratory nature of this study, no formal hypothesis testing was conducted. Instead, findings were analyzed descriptively to assess trends in PRS utilization and their impact on physician decision-making.

For the qualitative analysis of physician feedback surveys, content analysis was performed to identify recurring themes regarding PRS usability and clinical utility. Open-ended survey responses were coded and categorized systematically to explore common patterns in physicians’ perceptions of the PRS-based risk profiles.

## 3 Results

### 3.1 Descriptive characteristics of the study population

A total of 150 participants were included in the study. Details of the descriptive characteristics of the study population are presented in Table 1. In brief, the mean age of the participants was 41.9 years, with 56% being male and 44% female. The average LDL-cholesterol (LDL-C) level was 106 mg/dL, while the mean HDL-cholesterol (HDL-C) level was 55 mg/dL. Regarding blood pressure measurements, the mean systolic blood pressure (SBP) was 120 mmHg, while the mean diastolic blood pressure (DBP) was 77 mmHg.

**TABLE 1:** Descriptive characteristics of the study population.

Characteristic	N = 150
Females, n (%)	66 (44%)
Age (years), mean (sd)	41.9 (10.3)
LDL-C (mg/dL), mean (sd)	106 (25)
HDL-C (mg/dL), mean (sd)	55 (19)
SBP (mmHg), mean (sd)	120 (13)
DBP (mmHg), mean (sd)	77 (9)
CAD PRS, mean (sd)	40.7 (25.3)

### 3.2 PRS utilization in clinical decision-making

**FIGURE 2:**
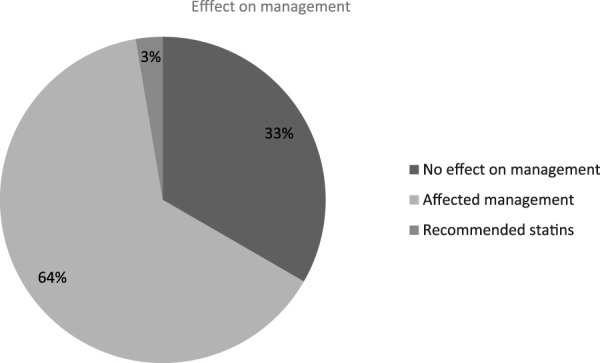
PRS effectiveness on physician decision-making.

PRS results influenced physician decision-making in 100 out of 150 cases (67%), while in the remaining 50 cases (33%), PRS findings did not appear to alter the initial treatment approach. Among the 100 cases where PRS played a role, physicians modified their management strategy in 96 cases (64%), and in 4 cases (3%), PRS led to a specific recommendation for commencing statin therapy (Figure 2).

### 3.3 Physician recommendations based on PRS results

In the 100 cases where PRS influenced clinical decisions, physicians provided a range of recommendations, including patient education, lifestyle modifications, enhanced monitoring, and pharmacologic interventions (Table 2).

**TABLE 2:** Physician recommendations based on PRS assessment.

Physician recommendation	n
Education/Raise Awareness	73
Lifestyle Modifications	6
Increased Patient Monitoring	6
Reassured Current Treatment Course	5
Recommended Statin Therapy	4
Considered Intensified Statin Therapy	2
Referred for Coronary CTA	1
Other	3

The most frequent recommendation was raising patient awareness, recorded in 73 cases. Notably, in 48 of these cases, physicians explicitly used the PRS system as an interactive educational tool to facilitate discussions with patients regarding their genetic risk and their implications for CAD prevention. By integrating PRS data into patient consultations, physicians aimed to enhance patient understanding of their cardiovascular risk profile and encourage enhanced proactive health management.

Lifestyle modifications were reported as recommended in six cases, focusing on dietary changes, smoking cessation, and increased physical activity.

In another reported six cases, increased patient monitoring was advised, particularly for individuals with high PRS values for CAD or associated risk factors (e.g., LDL, hypertension, diabetes). Physicians recommended more frequent lipid panels, blood pressure monitoring, or periodic HbA1c testing to track risk factors over time.

In five reported cases, physicians reassured patients that their current treatment approach remained appropriate despite their genetic risk, emphasizing that no immediate changes were required.

PRS findings led to statin-related recommendations in four particular cases. These patients were advised to initiate statin therapy based specifically on their genetic risk profile. In two additional cases, physicians discussed higher-dosed statin therapy, considering a more aggressive lipid-lowering approach.

Finally, a small subset of cases included referrals and targeted clinical discussions, such as coronary CTA referral, tightening blood pressure targets, and advising salt intake reduction for patients with relevant risk profiles.

## 4 Discussion

This study evaluated the impact of integrating a polygenic risk score (PRS) for coronary artery disease (CAD) into clinical decision-making, revealing significant insights into how physicians utilized this tool in patient care. The findings indicate that PRS influenced physician decisions in 67% of cases (100 out of 150 participants), demonstrating its substantial role in shaping clinical approaches. While the majority of these decisions involved enhancing patient education and risk awareness, PRS also contributed to adjustments in treatment strategies, lifestyle recommendations, and targeted monitoring. These findings underscore PRS as a valuable adjunct to conventional risk assessment tools, offering additional layers of stratification beyond traditional clinical markers.

The results align with prior studies demonstrating the potential utility of PRS in cardiovascular risk prediction. Several studies have reviewed the theoretical benefits of PRS for CAD, emphasizing its potential to refine risk stratification and improve clinical decision-making (Harst et al., 2021; Klarin and Natarajan, 2022; Marston et al., 2023). Another study has explored how PRS information is incorporated into physician decision-making, contributing to the growing body of evidence on the influence of genetic risk scores in clinical practice (Kerman et al., 2023).

Findings from previous research also support the importance of physician education and structured implementation of PRS in healthcare (Adeyemo et al., 2021; Hao et al., 2022; Slunecka et al., 2022). Guideline-based studies have emphasized key challenges in PRS adoption, particularly the need for standardized recommendations and improved clinician training (Visseren et al., 2021). The 2021 ESC Guidelines on cardiovascular disease prevention have not yet incorporated PRS as a routine risk assessment tool, reflecting the ongoing need for structured implementation in clinical practice (Visseren et al., 2021). Additionally, prior research has demonstrated that physicians frequently use PRS as a tool for patient education and risk communication, reinforcing our finding that PRS serves an important role in guiding discussions about cardiovascular health (Lewis et al., 2022). Other studies further emphasized the significance of physician familiarity with PRS. Although clinicians generally recognize their potential, challenges such as the complexity of interpretation may still limit their widespread application (Slunecka et al., 2022).

The integration of PRS within an interactive dashboard streamlined the incorporation of genetic risk data with clinical parameters, enhancing its accessibility in routine practice. This structured approach enabled physicians to efficiently interpret PRS results and integrate them into patient discussions, contributing to increased engagement and usability. Our findings suggest that an intuitive system for PRS presentation can support its adoption in real-world settings, addressing some of the barriers previously identified in clinical genomics. Prior research has highlighted the importance of clear and actionable reporting formats for PRS to optimize their utility in patient care (Pacyna et al., 2023), and our study reinforces this need by demonstrating physician engagement with an automated PRS system.

Our findings suggest that PRS has the potential to refine risk assessment by providing additional stratification beyond traditional clinical markers. Physicians frequently used PRS to guide conversations about cardiovascular risk, motivate patients to adopt lifestyle changes, and reinforce existing treatment plans. This aligns with prior literature indicating that PRS can serve as a tool for shared decision-making rather than a sole criterion for prescribing interventions. In four cases, statins were prescribed despite LDL levels being below 130 mg/dL, based solely on high PRS scores, highlighting how genetic risk influenced clinical decisions beyond standard lipid thresholds.

Despite the promising implications, our study has limitations. The relatively small sample size (150 participants) may limit generalizability. Additionally, physician interpretations of PRS likely varied, as no standardized framework for its application currently exists. This lack of standardization raises ethical concerns that have been widely discussed in literature, including the risk of misinterpretation, potential psychological distress to patients, or even inappropriate clinical decisions (Andreoli et al., 2024; Chapman, 2023; Palk et al., 2019). Given that our cohort included physicians with an interest in genetics, results may not fully represent broader clinical populations, where familiarity with PRS may be lower. Moreover, the absence of a control group limits the ability to draw definitive conclusions regarding causality. This study, however, was not intended to assess causal effects, but rather to explore clinical uptake, interpretability, and integration of PRS in daily workflow. In addition, the present study did not evaluate the predictive accuracy of the PRS-integrated platform, as this was beyond its intended scope. Future studies should assess how this approach compares quantitatively to established risk estimation tools in terms of discrimination and calibration. Furthermore, the relatively young age distribution of the study population may have limited the extent to which pharmacologic decisions, such as statin initiation, were observed. This age range was intentionally selected to evaluate PRS use in younger individuals, a group where traditional risk tools may underestimate long-term cardiovascular risk. Finally, although the PRS used in this study was derived from a large-scale multiethnic GWAS, we did not perform ancestry-specific analyses, and genetic ancestry was not explicitly incorporated into model interpretation.Moving forward, future research should focus on the development of clearer and more standardized guidelines for PRS interpretation and reporting. While some recommendations may already exist, their applicability and clarity in clinical practice remain limited (O'Sullivan et al., 2022). Establishing more specific guidance would help ensure consistent and effective use of PRS across different settings. Additionally, greater emphasis should be placed on educating physicians in the proper application of PRS. Without sufficient training, PRS integration may remain inconsistent and underutilized. Developing structured training programs, including workshops and decision-support tools, could help bridge this gap and optimize PRS implementation in routine cardiovascular care. Furthermore, the importance of patient use of these dashboards should not be overlooked, as it contributes to improved adherence to and understanding of physician-suggested CAD interventions.

In conclusion, this study provides empirical evidence on the real-world utilization of PRS in clinical practice, demonstrating its role in both physician decision-making and patient education. While PRS was primarily used to enhance risk awareness rather than directly alter pharmacologic management, its integration into cardiovascular risk assessment offers a promising avenue for personalized medicine. As genetic risk stratification continues to evolve, well-defined implementation strategies will be critical in ensuring that PRS translates into cost effective and meaningful improvements in cardiovascular disease prevention and for patient outcomes.

## Data Availability

The raw data supporting the conclusions of this article will be made available by the authors, without undue reservation.
